# Use of Optical Coherence Tomography and Electroretinography to Evaluate Retinal Pathology in a Mouse Model of Autoimmune Uveitis

**DOI:** 10.1371/journal.pone.0063904

**Published:** 2013-05-14

**Authors:** Jun Chen, Haohua Qian, Reiko Horai, Chi-Chao Chan, Rachel R. Caspi

**Affiliations:** 1 Laboratory of Immunology, National Eye Institute, National Institutes of Health, Bethesda, Maryland, United Sates of America; 2 Vision Function Core, National Eye Institute, National Institutes of Health, Bethesda, Maryland, United Sates of America; Justus-Liebig-University Giessen, Germany

## Abstract

Experimental autoimmune uveoretinitis (EAU) in mice is a model for human autoimmune uveitis. Longitudinal follow-up is only possible by non-invasive techniques, but the information obtained by visual fundus examination can be limited. We therefore evaluated the efficacy of optical coherence tomography (OCT) and electroretinography (ERG) to monitor pathological and functional changes of the retina in vivo. OCT imaging and ERG recording as a measure of visual function were compared with visual fundoscopic imaging and histology findings in the same mouse. Our results showed that OCT imaging of the retina was well correlated with clinical and histological observations in mice during EAU. However, OCT imaging was more sensitive than fundoscopic imaging in detecting the cell infiltrates at the early phase of disease onset. Furthermore, by allowing multi-layer cross- and horizontal-sectional visualizations of retinal lesions longitudinally in a noninvasive fashion, OCT added information that could not be obtained by fundoscopic and histological examinations. Lastly, retinal thickness obtained by OCT imaging provided a key indicator reflecting disease activity, which showed a close association with visual dysfunction as measured by ERG recordings in EAU mice. Thus, our findings demonstrate that OCT is a highly sensitive and reliable technique, and a valuable method for the semi-quantitative evaluation of retinal inflammation in vivo in the mouse.

## Introduction

Experimental autoimmune uveoretinitis (EAU) is an animal model of autoimmune uveitis that resembles human uveitis [1]–[3]. EAU is characterized by retinal and choroidal inflammation, vasculitis, photoreceptor destruction, and loss of vision [4]. EAU can be induced in mice by active immunization with retinal antigens in complete Freund adjuvant (CFA), including interphotoreceptor retinoid-binding protein (IRBP), retinal arrestin, or by adoptive transfer of autoreactive T cells specific to retinal antigens [1], [5]. The murine model of EAU has notable similarities to the spectrum of clinicopathological features of human uveitis and serves as preclinical model for studying immunopathogenesis and translation of immunotherapies [6].

To quantify the extent and severity of disease, *in vivo* longitudinal studies of the pathological and functional changes during EAU are essential for monitoring the onset and progress of disease during therapeutic intervention. The approaches routinely used to date consist of clinical scoring by standard fundoscopy supplemented by digital fundus imaging, and ex vivo histological scoring. Clinical EAU assessment involves examination of the retina of live mice using a fundus microscope [7], aided by fundus photography, limited to 2D-view images for disease assessment [8]. Histology relies on eye sections in samples collected post mortem, so by definition does not lend itself to an in vivo longitudinal study of the same individual.

A number of studies have indicated that optical coherence tomography (OCT) has becoming a well-established state-of-the-art imaging tool in ophthalmology [9]–[11]. OCT can be regarded a type of “optical biopsy”, providing information on retinal pathology in situ and in real time, with resolutions approaching that of excisional biopsy and histopathology. Up to now, OCT has been useful for serial observation of different retinal diseases in patients as, e.g., age related macular degeneration, glaucoma, posterior uveitis and birdshot chorioretinopathy [12]–[15]. However, the correlation of in vivo OCT imaging with ex vivo histological findings in patient eyes may not be available for obvious reasons. In various animal studies, OCT was employed as a novel method for real-time imaging of retina *in vivo* in degenerative diseases [16], [17]. Although a recent report briefly describes the application of OCT in rat EAU [18], a correlation with functional vision parameters was not performed.

Because the rat is increasingly being supplanted by the mouse as an EAU model, we decided to embark on a study to correlate OCT with clinical, histological and functional parameters in murine EAU. Using a Bioptigen Envisu R2200 ultra-high resolution OCT system to obtain and quantify high-resolution morphological sections of the mouse retina, we compared horizontal and cross-sectional OCT image with fundus images and histology sections. Visual function was evaluated by electroretinography (ERG). Our data demonstrate that the wide range of retinal pathology detected by OCT correlates well with histological sections and fundus imaging. Importantly, the three-dimensional imaging allows to quantitate retinal thickness (RT) as an additional measure of disease activity and to detect the vitreal cellular infiltrates at an early phase of inflammation with higher sensitivity in comparison to standard fundoscopy and fundus imaging. Finally, with the ability to document development of retinal pathology in individual animals over time, OCT can reduce the numbers of animals needed for the study.

## Materials and Methods

### Animals

B10RIII mice purchased from the Jackson Laboratory were maintained under specific-pathogen free conditions and treated in accordance with the ARVO Statement for the Use of Animals in Ophthalmic and Vision Research under the animal study protocol approved by the Animal Care and Use Committee of the NIH.

### Induction of EAU and assessment of disease by fundoscopy

Induction of EAU by active immunization with 6–8 µg IRBP emulsified in complete Freund's adjuvant (CFA; Sigma, St. Louis, MO) as described previously [19]. Eyes were examined for clinical signs of uveitis using a binocular fundus microscope with coaxial illumination. Mice were anesthetized systemically by intraperitoneal injection of ketamine (77 mg/kg) and xylazine (4.6 mg/kg) and ocular surface was anesthetized by 0.5% Alcaine drops. The pupils were dilated using 0.5% Tropicamide and 0.5% phenylephrine hydrochloride. A drop of sterile physiological solution was placed on the cornea and a microscope coverslip on the cornea served as a lens to equalize refraction. Eyes were examined for engorged blood vessels, constricted blood vessels (“cuffing”), white linear lesions, subretinal hemorrhages, and retinal detachment. Clinical EAU score was evaluated on a scale of 0–4, as described in detail elsewhere [7], [20].

### Histology

Eyes were harvested at different time points after immunization, stained with standard hematoxylin and eosin and processed for histopathologic examination. The severity of EAU was evaluated in a masked fashion on a scale of 0–4 using previously published criteria based on the number, type, and size of lesions [4]. Eighteen mice were included for histological examination, and eyes of 2–3 mice were harvested at individual time point.

### Retinal imaging microscopy

Mice were anesthetized and their pupils dilated as for standard fundoscopy, above, and the fundus was imaged using the Micron II small animal retinal imaging AD camera (Phoenix Research Laboratories, INC).

### Spectral Domain Optical Coherence Tomography (SD-OCT) imaging

Mice were anesthetized and their pupils dilated as described above. Artificial tears (Systane Ultra, Alcon) were used throughout the procedure to maintain corneal moisture and clarity. SD-OCT images were obtained in mice developing uveitis using the Bioptigen Spectral Domain Ophthalmic Imaging System (SDOIS; Bioptigen Envisu R2200, North Carolina). Image acquisition software was provided by vendor. Both averaged single B scan and volume scans were obtained with images centered on optic nerve head. Thirteen mice comprising two repeat experiments were included for the longitudinal study.

### Assessment of retinal thickness

Retinal thickness was measured manually from each B-scan OCT image, approximately 1 optic disc diameter (∼50 µm) away from the both edges of the optic disc. Retinal thickness was measured and averaged from the intensity peak of boundary corresponding to the vitreo-retinal interface to the intensity peak corresponding to the retinal pigmented epithelium. The fold change of retina to baseline was calculated individually and is presented as Mean ± SEM.

### Electroretinography (ERG)

Retinal function was evaluated by recording of dark- and light-adapted ERG (Espion E2 System, Diagnosys LLC). Mice were dark adapted for overnight before ERG recording, and all procedures were performed under dim red light. Mice were anesthetized and their pupils dilated as described above. For the ERG recordings, electrodes were placed on the center of cornea. Reference and ground electrodes were attached to the mouth and placed subcutaneously in the neck-back region. The a-wave amplitude was measured from the baseline to the trough of the a-wave, and b-wave amplitude was measured from the trough of the a-wave to the peak of the b-wave. Each experimental group consisted of five to six mice.

### Statistical analysis

All data are expressed as mean ± SEM. Statistical analyses of EAU scores were done using the Mann-Whitney test. P<0.05 was considered statistically significant.

## Results

### Correlation of OCT with histological findings in the retina

**Figure 1:**
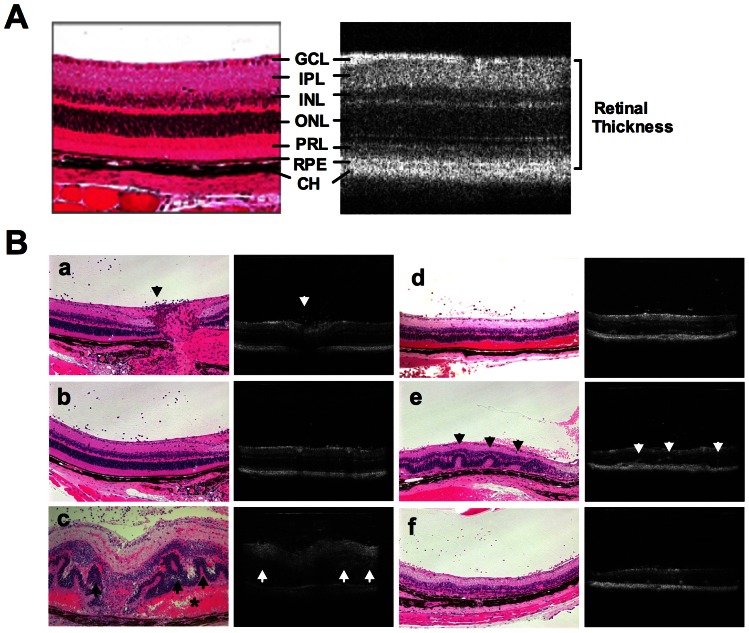
Comparison of OCT images with fundus and histological findings of the retina in EAU. EAU was induced in B10RIII mice by immunization with 8 ug IRBP in CFA. **A**, Normal retinal layers in a healthy eye assessed by cross-sectional OCT image in comparison with histological section of murine retina. Note ganglion cell layer (GCL), inner plexiform layer (IPL), inner nuclear layer (INL), outer nuclear layer (ONL), IS/OS of photoreceptor layer (PRL), retinal pigment epithelium (RPE) and choroid (CH). **B** (a–f), Comparison of cross-sectional OCT images of retina with histological sections of the same eyes at different stages of EAU: **a**–**b**, early onset of EAU (13 days post immunization). Note largely normal retinal morphology on OCT and histology, but a moderate degree of cellular infiltrates is apparent in the vitreous near the optic nerve head (arrow); **c**, acute phase of EAU (14 days post immunization). Note retinal pathology including vitritis, retinal edema, retinal folds (arrow), subretinal hemorrhage (asterisk), retinal and choroidal inflammation. Heavy cellular infiltration in the anterior chamber (not shown) and vitreous limit OCT resolution of retinal layers. In correlation with the pathological findings, OCT shows cellular infiltrates in the vitreous, retinal vasculitis and edema, and retinal folds; **d**–**f**, resolution phase of EAU (21–28 days post immunization), partial clearing of ocular media facilitate OCT visualization of cellular infiltration in the vitreous (d), retinal folds (e, arrow), choroiditis (e, yellow arrow) as well as degenerating PRL (f). Eighteen mice were included for histological examination, and eyes of 2–3 mice were harvested at individual time point.

We first performed a comparison of the same eyes using OCT and histological sections in normal and in EAU mice at various stages of disease (Figure 1). Each mouse was first subjected to fundoscopic examination and photography, next the retina was imaged by OCT, and finally the eyes were collected and processed for histopathology.

In healthy retina, cross-sectional OCT B-scan clearly resolved distinctive retinal layers, including ganglion cell/optic nerve fiber layer, inner plexiform layer, inner nuclear layer, outer plexiform layer, outer nuclear layer, inner/outer segments (IS/OS) of photoreceptor layer, RPE and choroid, corresponding to the same layers on histological section of the retina (Figure 1A).

To validate the utility of OCT to image retinal lesions in uveitic retina, we compared the cross-sectional OCT B-scan images with histological sections of the same eyes at different stages of disease (Figure 1B, subpanels a–f). At the early phase of EAU, on day 12–13 after immunization (a–b), OCT still showed a relatively normal appearance of retinal morphology, as did the histology assessment. A moderate degree of cellular infiltration was observed in the posterior vitreous near the optic nerve head, along with appearance of retinal edema. Disease developed very rapidly and within 24 h, on day 14, vitritis, retinal edema, retinal folds and infiltrates, subretinal hemorrhage, and choroidal inflammation became apparent (c). In correlation with the histopathological findings, OCT detected cellular infiltrates in the vitreous, retinal vasculitis and edema, and retinal folds. Due to heavy proteinaceous exudates and cellular infiltrate in the anterior segment (not shown) and the vitreous, that blocked the OCT signal, retinal layers could not be clearly distinguished. At post-peak phase of EAU on day 21–28 after immunization, ocular exudates diminished and OCT was able to distinguish discrete lesions, such as cellular infiltration in the vitreous (d), retinal folds (e), choroiditis (e) and loss of photoreceptor layers (f). Thus, OCT imaging showed a close correlation with the histological findings and non-invasively reflected the pathological changes in the retina during EAU.

### Correlation of OCT with fundus photography in the retina

**Figure 2:**
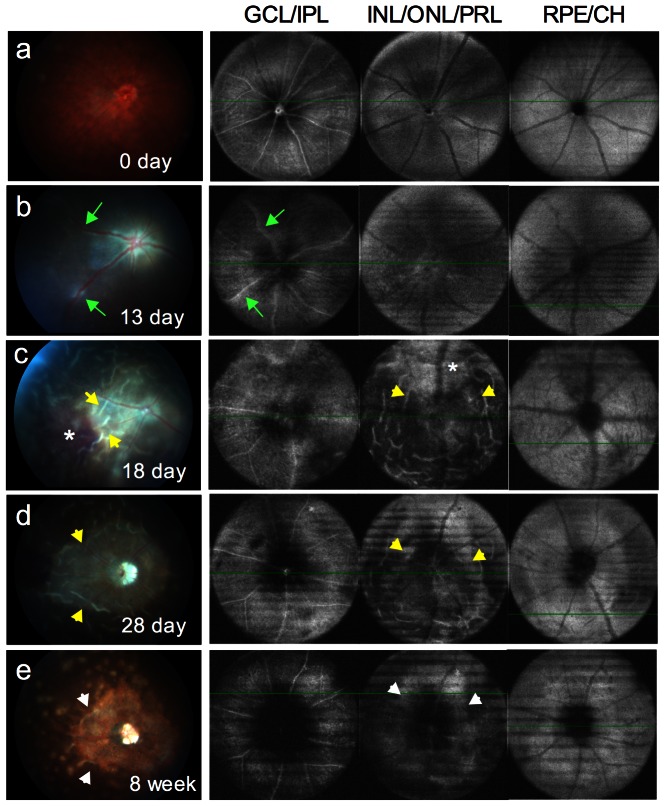
Comparison of OCT images with color fundus photographs. Horizontal-sectional 2D-view OCT images of GCL-IPL, INL-ONL-PRL, or RPE-CH in comparison with color fundus photographs at different stages of EAU: **a**, normal retinal structure; **b**, onset of EAU (13 days post immunization), horizontal-sectional OCT images showed swelling vessels and peri-vascular exudates (green arrows) in the layer of GCL-IPL; **c**, acute phase of EAU (18 days post immunization), peri-vascular exudates in GCL-IPL layer, retinal folds (yellow arrows) and subretinal hemorrhage (asterisk) seen in INL-ONL-PRL layer were detected by OCT 3D-view rectangle scanning; **d**, resolution phase of EAU (28 days post immunization), OCT detected a ring-like retinal thinning (yellow arrows) around the optic nerve head formed through INL-ONL-PRL; **e**, late phase of EAU (8 weeks post immunization), extensive ring-like retinal atrophy (arrows) in INL-ONL-PRL. Note parallel clinical features in the fundus images. Data are representative of 13 mice from two individual experiments.

Next, we compared the horizontal-sectional OCT volume scan images with the fundus images taken by Micron-II fundus camera in the same eye. 2D-view OCT images were captured in multiple retinal layers corresponding to (1) ganglion cell and inner plexiform layers, (2) inner/outer nuclear layers and IS/OS of photoreceptors layer, or (3) RPE and choroid layers. We compared these OCT images with standard fundus photographs in the same eye at different stages of EAU (Figure 2). OCT detected normal retinal structure in multiple layers at day 0 before immunization (a). In keeping with the fundus images, OCT exhibited engorged blood vessels and peri-vascular exudates (green arrows) in ganglion cell and inner plexiform layers at the early phase of E?U13 days post immunization (b). At the acute phase of EAU 18 days post immunization, similar to the fundus image, severe peri-vascular exudates in ganglion cell and inner plexiform layers, retinal folds (yellow arrows) and subretinal hemorrhage (asterisk) in inner/outer nuclear and IS/OS of photoreceptor layers were detected (c). At the resolution phase of EAU, 28 days post immunization, as shown in the fundus image, OCT detected a formation of ring-like retinal atrophy around the optic nerve head through inner/outer nuclear and IS/OS of photoreceptors layers (d). A ring-like degeneration in inner/outer nuclear and IS/OS of photoreceptors layers (arrows) was further confirmed by OCT at the late phase of EAU 8 weeks after immunization (e).

The results above reveal a close correlation of cross-sectional and horizontal-sectional OCT images with fundus images and histology sections of the same retina during EAU. More sensitive than in vivo 2D-view fundus imaging, OCT captures cross-sectional pathological lesion. These cross-sectional images detect the early onset of disease by visualizing cellular infiltrates in the vitreous and assess retinal edema by the change of retinal thickness. Moreover, more than histological sections, OCT captures horizontal-sectional images of the retina. The horizontal-sectional images give an overview of the pathological lesions present in the different retinal layers. These observations suggest that OCT imaging is a non-invasive and accessible tool for detection of retinal lesions during EAU.

### Longitudinal OCT imaging of the retina during the course of EAU

**Figure 3:**
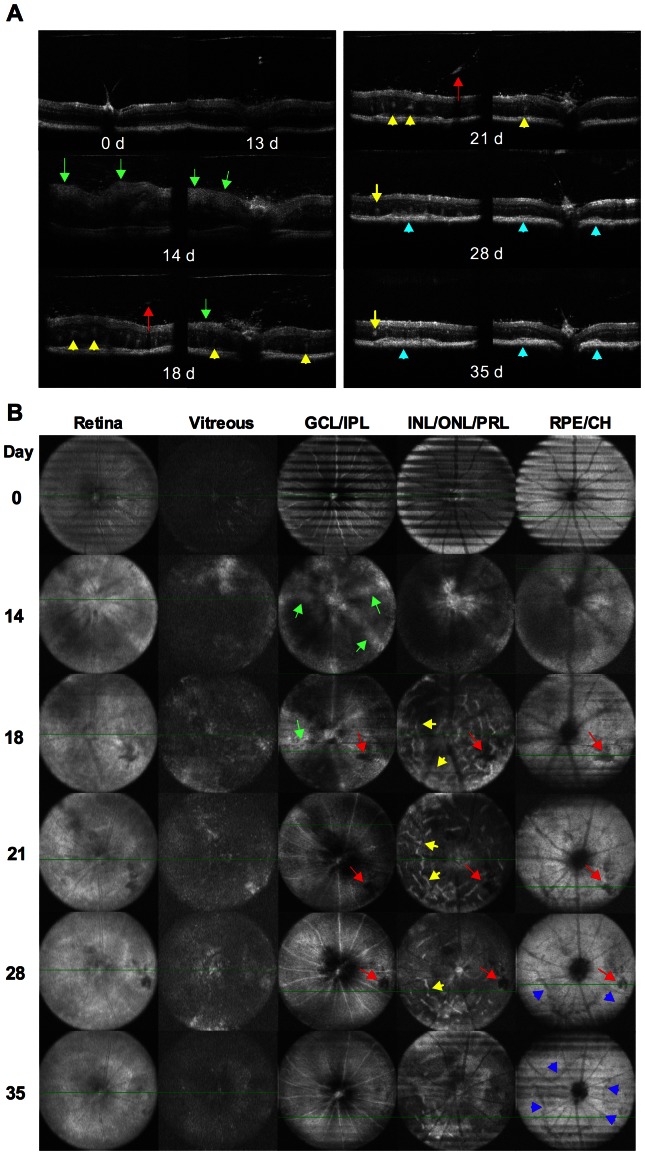
Longitudinal OCT imaging of the retina during the course of EAU. EAU was induced in B10RIII mice by immunization with 8 ug IRBP in CFA. Cross (A) and horizontal (B) sections of OCT images were captured at the indicated time points post immunization. **A**, Cross-section of OCT image showed normal retinal morphology before immunization for EAU (day 0). The retinal layers were slightly less distinguishable than normal at the pre-peak phase of disease (day 13 post immunization), when retinal edema with increase of retinal thickness appears along with few cell infiltrates around the optic nerve head. On day 14, low OCT signal was detected. Abnormal retinal architecture including perivascular exudates (green arrow) and retinal edema appeared. At 18–21 days after immunization, when the cell infiltrates were reduced in the vitreous, the OCT signal was partially restored. Prominent vasculitis (green arrow), retinal folds (yellow arrow), and vitreal hemorrhage (red arrow) were detected. At late phase of EAU, OCT revealed degenerating retinal layers (particularly in photoreceptor layer) with reduced retinal thickness, retinal folds, as well as choroid inflammation (blue arrow) that formed a ring like retinal lesion around the optic nerve in EAU mice on 28 and 35 days post immunization. **B**, Horizontal section of OCT images revealed pathological lesions in different retinal layers of the same eye. Data are representative of 13 mice from two individual experiments.

We next examined the utility of OCT as a longitudinal follow-up of the same eye during the course of EAU (Figure 3). Cross-sections of OCT B-scan images were obtained at different stages and shown in Figure 3A, and horizontal-sections of OCT volume scan obtained at multiple layers of the retina are depicted in Figure 3B. Normal retinal morphology is apparent before immunization for EAU (day 0). The retinal layers were slightly less distinguishable than normal at the pre-peak phase of disease 13 days post immunization, when retinal edema with increase of retinal thickness was detected by OCT along with few cell infiltrates around the optic nerve head. On day 14, OCT could not clearly resolve the retinal layers, due to partial blocking of the OCT signal by heavy proteinaceous exudation and cellular infiltration in the anterior and posterior segments of the eye. It should be pointed out that for illustration purposes, we chose the clearest OCT images obtained at this time point, realizing that they are not the most representative. It was nevertheless possible to detect an increase in retinal thickness by OCT and a clearly abnormal retinal architecture including perivascular exudates (green arrow). At 18–21 days after immunization OCT detected prominent retinal vasculitis (green arrow), retinal folds (yellow arrow), and vitreal hemorrhage (red arrow). At 28 days post immunization, when the cell infiltrates were reduced in the vitreous and retinal edema disappeared, the OCT signal was restored. OCT revealed degenerating retinal layers (particularly in photoreceptor layer) with reduced retinal thickness, retinal atrophy, as well as retinal and choroid inflammation (purple arrow) in EAU mice on 28 and 35 days post immunization. Ring-like retinal atrophy adjacent to the optic nerve in inner/outer nuclear and IS/OS of photoreceptors layers and in RPE and choroid layers were also detected and were confirmed by OCT (blue arrows). The longitudinal disease course and pathological changes in the retina are summarized in Table 1.

**Table 1:** Morphologic and functional changes of retinal lesions distinguished by OCT imaging, fundus photography, histology and ERG during EAU in B10RIII mice.

Disease stage & assessment	Day p.i.	Pathological and functional changes in the retina
***OCT***		
Healthy	0	Normal retinal morphology, clear retinal layers
Pre-peak	11–13	Cellular infiltrates around optic nerve head, retinal edema appears
Peak	14–15	Low OCT signal, abnormal retinal appearance, severe cellular infiltrates & exudates, retinal edema & increase in retinal thickness
Post-peak	18–21	Cellular infiltrates, retinal edema & increase in retinal thickness, retinal folds, vitreal hemorrhage
Late	28–35	Loss of photoreceptor layer & decrease in retinal thickness, choroiditis
***Fundus photo***		
Healthy	0	Normal retinal morphology
Pre-peak	11–13	Perivascular exudates and retinal edema appear
Peak	14–15	Poor signal due to opacity of the ocular media
Post-peak	18–21	Perivascular exudates, retinal edema & folds, vitreal hemorrhage
Late	28–35	Retinal atrophy, choroiditis
***Histology***		
Healthy	0	Normal retinal morphology, clear retinal layers
Pre-peak	11–13	Cellular infiltrates, retinal edema appears
Peak	14–15	Abnormal retinal appearance, severe cellular infiltrates & exudates, retinal edema & increase in retinal thickness
Post-peak	18–21	Cellular infiltrates, retinal edema & folds, vitreal hemorrhage
Late	28–35	Loss of photoreceptor layer & thinning of retina, choroiditis
***ERG***		
Healthy	0	Normal ERGs
Pre-peak	11–13	Decline of ERGs by 25%
Peak	14–15	Decline of ERGs by 90%
Post-peak	18–21	ERGs remain flat
Late	28–35	ERGs remain flat

EAU was induced in B10RIII mice by immunization with 8 µg IRBP peptide in CFA. The kinetics of disease onset and progress were visualized by OCT imaging, fundus photography, histology and ERG recording. The character of EAU at different phase of disease was summarized based upon above observation.

### Assessment of retinal thickness by OCT

Due to inherent limitations of the methods, neither fundus imaging nor fundus microscopy could provide quantitative information of retinal thickness for EAU scoring. We therefor employed OCT imaging to measure retinal thickness in comparison with disease severity observed by fundoscopy.

**Figure 4:**
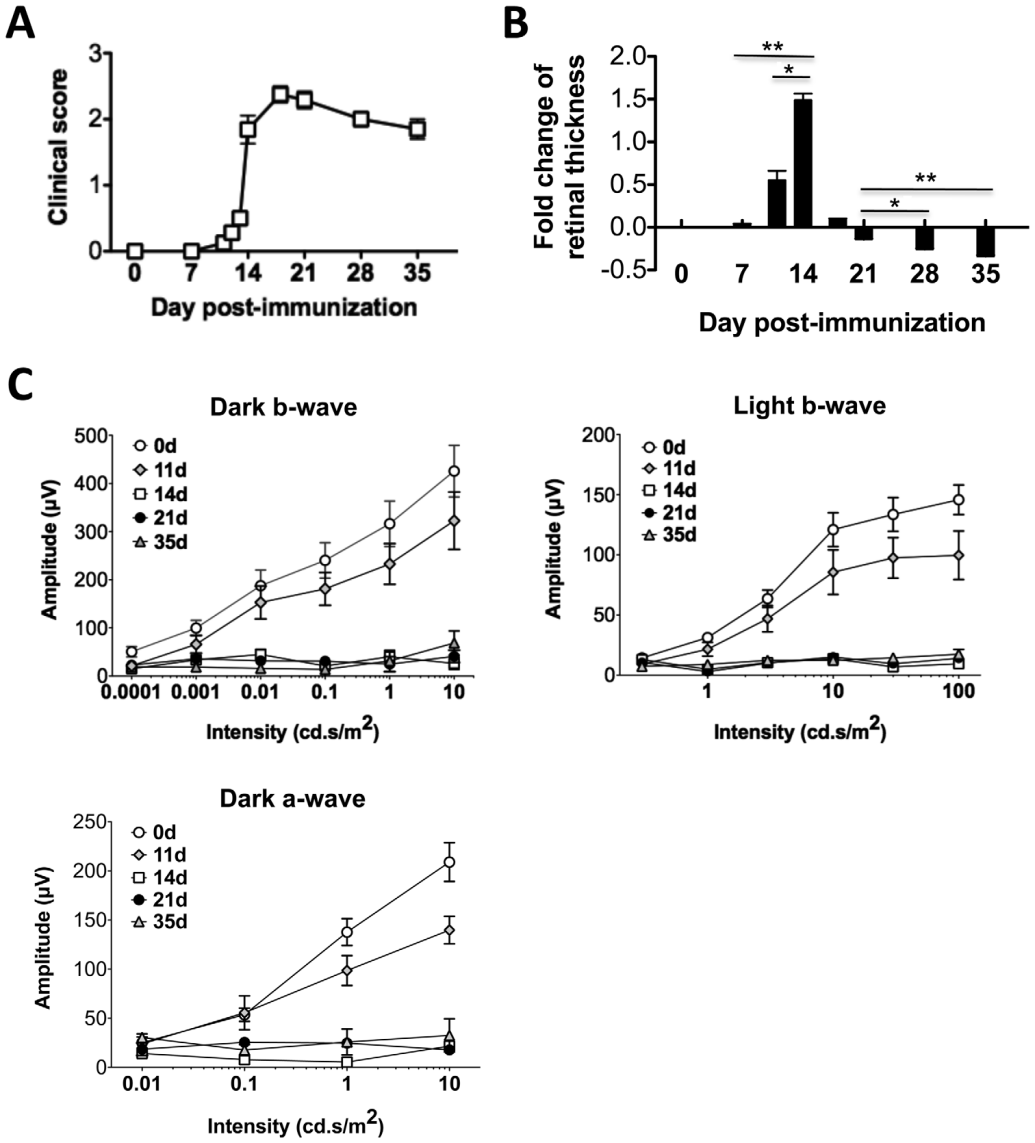
Serial ERG response after dark and light adaptation in comparison to disease severity and fold change of retinal thickness obtained by OCT imaging. EAU was induced in B10RIII mice by active immunization with IRBP161–180 in CFA. Mice were monitored and followed up at the indicative time points by standard fundoscopy, OCT and ERG. **A**, EAU clinical scores as determined by clinical examination using standard fundoscopy. **B**, Fold change of retinal thickness measured by OCT. **C**, Dark- and light-adapted ERGs during EAU. Data are present as mean ± SEM of 13 mice from two individual experiments. Statistical analyses of retinal thickness are performed using the Mann-Whitney test. *, p<0.05; **, p<0.01.

Clinical score of EAU was evaluated on a scale of 0–4, as described in elsewhere [5], [21] using an adapted funduscopic microscope. In B10RIII mice, EAU onset occurred 11–13 days post immunization. Following the acute inflammation on day 14–21 post-immunization, mice manifested progressive reduction of ocular inflammation and progressive retinal degeneration, with active disease ending around day 35 (Figure 4A). On day 14 post-immunization the disease scores were assigned as the best estimate, because of limited visibility as a result of the opacity of ocular media at that time point.

Retinal thickness at the different stages of disease was assessed based upon the cross-sectional OCT images obtained longitudinally in individual mice at different phase of disease, and the fold change of retinal thickness to baseline was measured and shown in Figures 4B. In keeping with the clinical observation, we detected a dramatic increase of retinal thickness (approximately half fold increment over baseline) at the pre-peak phase of disease (on 12–13 days post immunization), when cellular infiltrates appeared. A sharp increase of retinal thickness (∼1.5-fold over baseline) was detected at the peak of disease 14 days post immunization (p<0.05), and was followed by a decline on 21 days post immunization. Progressive reduction of retinal thickness was observed at the late phase of EAU, reaching nearly 0.4-fold reduction in retinal thickness on day 35 days post immunization (p<0.01).

The sharp increase of retinal thickness is well correlated with the severity of EAU shown by histology and fundus findings. The thinning of retina in association with photoreceptor loss and retinal degeneration was further confirmed by histological examinations. Based upon the information described above, we consider retinal thickness as a clinical indicator for measuring disease activity and retinal degeneration in EAU mice.

### Assessment of retinal function by ERG during EAU

To examine whether and when retinal inflammation of EAU lead to functional changes in vision, we employed an ERG recording system to evaluate the visual function in mice with EAU (Figure 4C) in comparison with retinal pathology findings obtained by OCT and fundus imaging and histology examination. We followed up both scotopic (dark-adaptation) and photopic (light-adaptation) ERG responses in EAU mice during the course of disease. No reduction in ERG amplitudes was detected on day 7 post immunization. EAU development led to a detectable reduction in both dark- and light-adapted b- and a-wave amplitudes on 11 days post immunization when early ocular inflammation was detectable and retinal edema reached 0.5-fold increment in thickness over baseline. By day 14 post immunization, when retinal edema reached 1.5-fold of baseline the b- and a-wave amplitudes in response to both dark and light adaptations dropped sharply by 90% and did not return to normal during the course of EAU or later after disease resolution. Although scotopic and photopic ERGs reflect retinal cell activities of different signal pathways, such as rod- and cone-mediated visual signals, we found the reduction of ERGs in response to either dark or light adaptation exhibited in a similar pattern during EAU. These functional changes are reflected in corresponding pathological lesions, OCT findings and fundus changes (Table 1). In mouse model of EAU in B10RIII mice, our ERG study show that vision function loss is associated with a dramatic increase of retinal thickness and structural change of retina. In addition, retinal thickness dropped below baseline as inflammation subsided, in line with the photoreceptor atrophy seen on histology.

## Discussion

In the present study, we evaluated EAU using a Bioptigen OCT system to non-invasively visualize and quantify high-resolution morphological sections of the mouse retina, and compared with fundoscopy and histology. Using OCT, we were able to detect a wide range of retinal pathology and to monitor the disease longitudinally throughout the course of EAU. The results compared well to histological sections, clinical score and fundus images of the eyes. We conclude that OCT imaging is a reliable method that is amenable to standardized imaging for the semi-quantitative clinical evaluation of retinal inflammation in mice.

Comparison of results from different laboratories can be difficult, since clinical grading is subjective and scoring criteria can vary among research groups. Several studies in the past have described an image-based clinical grading system for semi-quantitative evaluation of the severity of retinal inflammation in mouse model of EAU [8], [21] using fundus photography. However, fundus photography is limited by the technology of 2D imaging and by inadequate ability to accurately measure pathological lesions, such as cellular infiltrates, edema and structural damage to the retina, which are important indicators for disease scoring. In our experience, OCT has three distinct advantages as a noninvasive method to follow disease development longitudinally, that are not achieved by fundus examination. First, compared to fundoscopy and digital fundus imaging it can detect subtle changes *in vivo* by cross-sectional images (as well as histology section) during the pre-peak phase of disease, such as mild cellular infiltration near the optic nerve and retinal edema. Second, compared to cross-sectional histology sections, OCT can visualize pathological lesions in different retinal layers in either cross-sectional or horizontal-sectional view. Third, OCT is able to discern retinal lesions and accurately measures retinal thickness, thus adding a valuable dimension to morphology and functional assessments of EAU. As with any imaging systems, there is technical limitation for OCT to distinguish individual retinal layers during the peak phase of disease (particularly on day 14 post-immunization). At that time, OCT signal is incapable of resolving ocular structure because it is partially blocked due to opacity of the ocular media.

OCT measurement of retinal thickness is utilized widely in clinical follow-up and diagnosis of ocular diseases, such as glaucoma [22], uveitis associated with multiple sclerosis [23], Vogt-Koyanagi-Harada disease [24], and macular edema [25]. We demonstrate that in addition to being an alternative to histopathology for visualizing ocular structures at peak disease, assessment of retinal thickness throughout the disease course provides a useful method to discriminate among the different stages of the disease in the mouse EAU model. Prominent retinal edema along with extensive structural damage of retina at the acute stage of EAU was correlated with rapid loss of visual function by ERG, whereas thinning of the retina correlated with resolution of inflammation and extensive photoreceptor loss, as appreciated by fundoscopy and by histopathology.

Our observation that visual function by scotopic and photopic ERGs was rapidly lost upon onset of inflammation (partially as early as day 11 and almost completely by day 14) raises the question whether the inflammatory mediators produced at that time by inflammatory cells might interfere with visual signal transduction, independently of actual physical damage to the photoreceptors. An answer to this question is of interest, but would require a very different set of experimental approaches and is beyond the scope of the current study.

The utility of OCT for imaging retinal lesions in the rat EAU model was recently reported, although the scope of that study was quite limited [18]. Our current study, besides being the first evaluation by OCT in the mouse EAU model, is also the first to perform a comprehensive comparison of anatomy, pathology and functional changes in the same eyes and in the same individual mice during the course of inflammation. Our study also complements and extends ERG studies of EAU in rats [26], [27] and more recently in mice [28]. Since mouse model of EAU is widely used as an animal model for studying human posterior and panuveitis: our findings bring valuable new insights into the pathological process of mouse EAU and provide a useful tool for evaluation of translational therapy using this model.
